# Dendriform pulmonary ossification in coronavirus disease 2019 pneumonia

**DOI:** 10.1590/0037-8682-0621-2021

**Published:** 2022-03-14

**Authors:** Pedro Paulo Teixeira e Silva Torres, Marcelo Fouad Rabahi, Edson Marchiori

**Affiliations:** 1 Multimagem Diagnósticos, Goiânia, GO, Brasil.; 2 Hospital Geral de Goiânia, Goiânia, GO, Brasil.; 3 Universidade Federal de Goiás, Goiânia, GO, Brasil.; 4 Universidade Federal do Rio de Janeiro, Rio de Janeiro, RJ, Brasil.

A 54-year-old man presented with severe acute respiratory syndrome coronavirus-2 (SARS-COV-2) positivity after experiencing flu-like symptoms. His condition progressed to acute respiratory failure, and he was placed on mechanical ventilation. Chest computed tomography (CT) showed bilateral ground-glass opacities with mild reticulation (Figure 1A). The patient was treated with supportive measures, including antibiotic therapy and corticosteroid therapy. He recovered well and was discharged. Two months later, he presented with worsening dyspnea and cough. A new CT showed improvement in the ground-glass opacities but a progression of the reticular opacities, with traction bronchiectasis on the anterior aspect of the superior lobes. Interstitial calcifications compatible with dendriform pulmonary ossification (DPO) were observed over the reticular abnormalities (Figure 1B-D). Steroid treatment improved the patient’s overall clinical condition.

**FIGURE 1:**
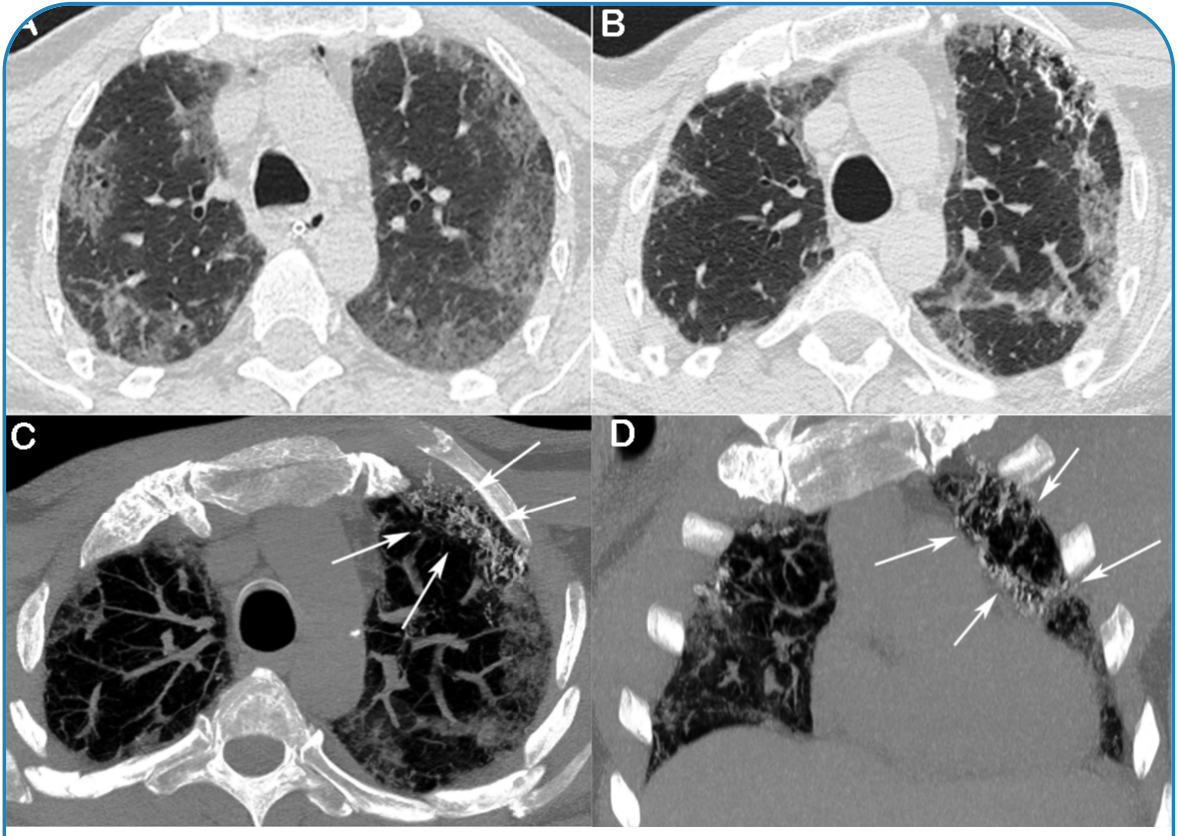
Axial computed tomography (CT) performed with lung window settings showed bilateral ground-glass opacities with mild reticulation (A). CT performed two months later demonstrated improvement of the ground-glass opacities but progression of the reticular opacities, with traction bronchiectasis on the anterior aspect of the superior lobes (B). Axial and coronal reconstructions acquired with mediastinal window settings showed interstitial calcifications compatible with dendriform pulmonary ossification over the reticular pattern (C and D,arrows).

Diffuse pulmonary ossification is a rare chronic process characterized by the formation of small mature bone fragments in the pulmonary parenchyma. It is classified as nodular or dendriform, with the former usually occurring in the context of chronic congestion. DPO is an interstitial process that occurs in the setting of fibrosing interstitial lung disease. It may evolve with osseous metaplasia, which is seen as nodular and branching calcifications in imaging studies. This pattern has been observed mainly in areas of reticulation rather than in honeycombing^1^ ^-^ ^3^.

Another condition recently related to DPO is cicatricial organizing pneumonia. This distinctive form of organizing pneumonia may manifest as persistent linear opacities that mimic fibrosing interstitial pneumonia. It may also be displayed as ossification foci in imaging and pathology studies^2^. Our case demonstrates the manifestation of extensive DPO, probably related to cicatricial organizing pneumonia, after coronavirus disease 2019 pneumonia.

## References

[1] Moreno BG, Weiland GB, Alegre MLS, Rodríguez JEV (2021). Accelerated Pulmonary Ossification as a Sequela of SARS-CoV-2 Pneumonia. RadiolCardiothorac Imaging.

[2] Saeedan MB, Farver C, Mehta AC, Yadav R (2019). Cicatricial Organizing Pneumonia with Dendriform Pulmonary Ossification: An Unusual Cause for a Recurrent Pneumothorax. Case Rep Pulmonol.

[3] Egashira R, Jacob J, Kokosi MA, Brun AL, Rice A, Nicholson AG (2017). Diffuse Pulmonary Ossification in Fibrosing Interstitial Lung Diseases: Prevalence and Associations. Radiology.

